# Etiological classification of depression based on the enzymes of tryptophan metabolism

**DOI:** 10.1186/s12888-014-0372-y

**Published:** 2014-12-24

**Authors:** Katsuhiko Fukuda

**Affiliations:** Soka Clinic of Psychosomatic Medicine, Fujimoto Bld. 4 F, 2-18-16 Takasago, Soka, Saitama, 340-0015 Japan

**Keywords:** Depression, Serotonin, Kynurenine, Tryptophan hydroxylase, Tryptophan 2,3-dioxygenase, Indoleamine 2,3-dioxygenase, Kynurenine-3-monooxygenase, Cybernetics

## Abstract

**Background:**

Viewed in terms of input and output, the mechanisms of depression are still akin to a black box. However, there must be main pivots for diverse types of depression. From recent therapeutic observations, both the serotonin (5-HT) and kynurenine pathways of tryptophan metabolism may be of particular importance to improved understanding of depression. Here, I propose an etiological classification of depression, based on key peripheral and central enzymes of tryptophan metabolism.

**Discussion:**

Endogenous depression is caused by a larger genetic component than reactive depression. Besides enterochromaffin and mast cells, tryptophan hydroxylase 1 (TPH1), primarily expressed in the gastrointestinal tract, is also found in 5-hydroxytryptophan-producing cells (5-HTP cells) in normal intestinal enterocytes, which are thought to essentially shunt 5-HT production in 5-HT-producing cells. Genetic studies have reported an association between TPH1 and depression, or the responsiveness of depression to antidepressive medication. Therefore, it is possible that hypofunctional 5-HTP cells (reflecting TPH1 dysfunction) in the periphery lead to deficient brain 5-HT levels. Additionally, it has been reported that higher TPH2 expression in depressed suicides may reflect a homeostatic response to deficient 5-HT levels. Subsequently, endogenous depression may be caused by TPH1 dysfunction combined with compensatory TPH2 activation. Reactive depression results from life stresses and involves the hypothalamic-pituitary-adrenal axis, with resulting cortisol production inducing tryptophan 2,3-dioxygenase (TDO) activation. In secondary depression, caused by inflammation, infection, or oxidative stress, indoleamine 2,3-dioxygenase (IDO) is activated. In both reactive and secondary depression, the balance between 3-hydroxykynurenine (3-HK) and kynurenic acid may shift towards 3-HK production via kynurenine-3-monooxygenase (KMO) activation. By shifting the equilibrium position of key enzymes of tryptophan metabolism, the classical classification of depression can be reorganized, as below.

*Peripheral classification of depression by key enzymes*

- TPH1 dysfunction
- TDO activation
- IDO activation

*Central classification of depression by key enzymes*

- TPH2 activation
- KMO activation

**Summary:**

Etiological classification of depression expressed by peripheral (TPH1, TDO, IDO) and central (TPH2, KMO) enzymes of tryptophan metabolism may enable depression to be viewed as a clear box, with the inner components available for inspection and treatment.

## Background

Historical classification of depression into “reactive” (or “exogenous”) and “endogenous” forms has produced no remarkable progress in its clinical picture or long-term outcome. This may be because of dynamic reciprocal interaction between life events and the genesis of depression, which can be observed from sociological, psychological or biological points of view [1,2].

Even though depression is characterized as an episodic illness, prospective studies have found that recurrence is the norm rather than the exception [3-5].

Furthermore, consistent evidence supports a “kindling hypothesis”, in which depressive episodes are more easily triggered over time, as a process that occurs by lowering the impact threshold for stressful life events (i.e., sensitization to minor events) or by increasing spontaneous dysregulation, both indicating progressive effects of depression [6-8]. Analysis of recurrence risk in a large twin study also suggested a genetic contribution, as patients with high genetic risk are “prekindled”, and show lower association between stressful life events and onset of depressive episodes compared with patients of low genetic risk [9].

It has been reported that an imbalance between glucocorticoid and mineralocorticoid receptors in depression, along with high-density glucocorticoid receptors, may contribute to hippocampal susceptibility to neuronal damage [10-12]. Subsequent hippocampal atrophy may result in further neuroendocrine dysfunction that includes the hypothalamic-pituitary-adrenal axis (HTPA axis) [13].

As a result of elevated glucocorticoids and compromised hippocampal functioning, downregulation of glucocorticoid receptor sensitivity may occur and further perpetuate metabolic and neuroendocrine disruption [14-16].

In these biological processes, stress and genetic vulnerability elevate glucocorticoid steroids and alter cellular plasticity via downregulation of growth factors and receptor sensitivity [17]. Reduction in growth factors, such as brain-derived neurotrophic factor (BNDF), impacts negatively upon structural and functional processes within the limbic system, especially the hippocampus. Chronic and recurrent depression may result in subsequent atrophy and further disruptions in neurocircuitry.

In 1980, DSM-III revisions created a new taxonomy of depression, and rather than exogenous and endogenous, depression was classified as “major” or “minor”, with no reference to etiology and only the diagnostic modifier “melancholic features” (despite inconsistent inter-rater reliabilities between melancholic and non-melancholic features) [18].

Nowadays, clinical psychiatry approaches depression as a cluster of maladaptive thoughts and behaviors requiring corrective treatment, without consideration of the heterogeneity and etiological complexity of the disease. Classification of depression is controversial, causing much debate among psychiatrists, as diagnoses are based on the presence of certain arbitrarily defined symptoms [19-22]. Different types of depression will have distinct causes and outcomes, and respond differently to treatment. At present, the biological mechanisms of depression are still akin to a black box, viewed merely in terms of input and output. However, there must be main pivots for diverse types of depression.

The effects of antidepressant treatment on the serotonin (5-HT) system may be intimately related to their therapeutic effect in major depression. As identified in the landmark Sequenced Treatment Alternatives to Relieve Depression (STAR*D) study, up to one-third of people with major depression do not respond to multiple antidepressant treatment attempts [23]. Conversely, the effect of the NMDA receptor antagonist, ketamine, on treatment-resistant depression appears both quick and substantial. Kynurenic acid (KYNA) is an endogenous tryptophan-derived compound that also blocks NMDA receptors. In the trial of Murrough *et al*., more drug-refractory patients responded to ketamine than the benzodiazepine midazolam [24]. However, approximately half of those who responded to ketamine relapsed over the next week. The antidepressant properties of the non-competitive NMDA receptor antagonist, MK-801 (dizocilpine), and the competitive NMDA receptor antagonist, CGP 37849 (DL-(E)-2-amino-4-methyl-5-phosphono-3-pentonoic acid) and its (R)-enantiomer CGP 40116, were studied in a chronic mild stress model of depression by Papp and Moryl [25]. In the study, animals were subjected to a variety of mild stressors for prolonged periods of time, showing substantially decreased consumption of palatable sucrose solution (anhedonia). They also found using this model that the stress-induced deficit in sucrose intake was gradually reversed by treatment with these NMDA receptor antagonists. The magnitude of the effect and its time course are comparable to that following similar administration of imipramine. Increased sucrose intake following chronic administration of imipramine and NMDA receptor antagonists is specific to stressed animals. Li *et al*. used a rat 21-day chronic unpredictable stress model to test the rapid action of NMDA receptor antagonists on depressant-like behavior. They found that chronic unpredictable stress exposure decreases expression levels of synaptic proteins, spine number, and frequency and amplitude of synaptic currents (excitatory postsynaptic currents) in layer V pyramidal neurons of the prefrontal cortex, and these deficits are rapidly reversed by ketamine [26]. Overall, these findings suggest that NMDA receptor antagonists may have antidepressant properties. Moreover, several lines of evidence indicate that chronic stress and BNDF downregulation are key components of depression pathology. Evidence from animal models of depression demonstrates that chronic stress impairs BNDF expression, and both antidepressant drugs effecting the 5-HT system and NMDA receptor antagonists correlate with increased BDNF synthesis and activity [27,28]. These observations suggest that both the 5-HT and kynurenine pathways of tryptophan metabolism may be of particular value to better understanding depression. Here, I propose an etiological classification of depression based on key peripheral and central enzymes of tryptophan metabolism.

## Discussion

The first step in reclassifying depression must consider the historical classification (including exogenous and endogenous depression), despite the lack of any outstanding differences between dynamic and mixed depressive states in terms of clinical picture and long-term outcome. Then, a new classification of depression can be considered by restructuring the historical classification.

Depression can be classified into two new forms using tryptophan metabolism: peripheral classification by key enzymes, or central classification by key enzymes.

### Classical etiological classification of depression

First, it is important to consider the physiological conditions vulnerable to depression.

Tryptophan is an essential amino acid obtained from dietary sources. Central tryptophan availability mainly depends on competition by large amino acids for transport across the blood–brain barrier (BBB). A large body of evidence supports association of exercise with increased plasma tryptophan and decreased plasma levels of the branched chain amino acids (BCAAs), leucine, isoleucine, and valine [29-33]. BCAAs inhibit tryptophan transport into the brain, therefore increased plasma tryptophan and decreased BCAAs cause a substantial increase in tryptophan availability to the brain. Patients with diets poor in tryptophan may be vulnerable to depression, as this essential amino acid is not naturally abundant, even in protein-rich foods. Moreover, individuals with sarcopenia or who do not exercise are also vulnerable to depression.

In the following section, I discuss the classical etiological classification of depression with potential involvement of tryptophan metabolism.

### • Endogenous depression

“Endogenous” (occurs from within) implies there is no discernible cause for the depression, and it is caused by a larger genetic component than reactive depression. This form of depression is characterized by a high proportion of biological symptoms (sleep and appetite disturbance, poor concentration and memory, loss of interest in sex).

#### Tryptophan metabolism can account for the etiology of this depressive state:

Two isoforms of tryptophan hydroxylase (TPH) have been discovered: TPH1, primarily expressed in the gastrointestinal tract, and TPH2, expressed exclusively in neuronal cells. Excluding the brain, large amounts of 5-HT are produced in enterochromaffin (EC) and mast cells, and stored in platelets. TPH1 expression was expected to be confined to EC and mast cells in the intestine. Unexpectedly, TPH1 was also found in 5-hydroxytryptophan-producing cells (5-HTP cells) in normal enterocytes lining the small intestine epithelium. 5-HTP cells are differentiated mucosal villous epithelial cells, expressing positive TPH1 staining without 5-HT immunoreactivity, and secreting intermediate 5-HTP into the mesenteric vein circulation, as stored 5-HT is not identified within them [34-36]. 5-HTP is regarded as a precursor of 5-HT, and 5-HTP cells are essentially thought to shunt 5-HT production in 5-HT-producing cells (5-HT cells), in both the periphery and brain.

TPH1 is associated with depression or the responsiveness of depression to antidepressive medication, by a considerable number of genetic studies [37-42]. There are two cellular locations for TPH1 dysfunction related to genetic studies: TPH1 dysfunction in EC and mast cells, or 5-HTP cells. If TPH1 dysfunction occurs in EC and mast cells, it may lead to decreased peripheral 5-HT, which cannot cross the BBB, therefore this situation is unlikely to be connected to 5-HT depletion in the brain. Consequently, TPH1 dysfunction in 5-HTP cells should be exclusively considered. Owing to its ability to ameliorate 5-HT depletion, 5-HTP is better than a placebo at alleviating depression [43]. Peripheral 5-HTP cells provide an endogenous substrate for L-aromatic amino acid decarboxylase in 5-HT cells, and it is possible that hypofunctional 5-HTP cells (reflecting TPH1 dysfunction) in the periphery lead to deficient brain 5-HT levels, and may be a factor for depression. Hyperfunction of 5-HTP cells may have the opposite effect, but hypofunction as a cause of pathology tends to be more common than hyperfunction. For example, the thyroid gland exhibits both hypothyroidism and hyperthyroidism, although the mean incidence of spontaneous hypothyroidism is greater than hyperthyroidism [44].

Conversely, there are also TPH2 abnormalities in depression. Bach-Mizrachi *et al.* reported that higher TPH2 expression in depressed suicides may reflect a homeostatic response to deficient brain 5-HT levels [45].

The genetic influence may decrease relatively if there is abundant tryptophan supply to the brain. It is possible that hypofunctional 5-HTP cells (reflecting TPH1 dysfunction) in the periphery, leading to deficient 5-HTP, low 5-HT, and concomitant compensatory TPH2 activation in the brain, may cause endogenous depression.

### • Reactive depression

#### • Secondary depression

“Reactive” implies the depression results from external stress occurring in the sufferer’s life. The risk of this type of depression is influenced more by the severity of life stresses than inherited factors. Symptoms are typified by worry and anxiety, and problems getting to sleep (rather than waking early in the morning, as in endogenous depression). It was originally argued that patients with reactive depression were less likely to respond to antidepressants.

In secondary depression, the depression is caused by a medical condition. One-third of patients with depression show elevated peripheral inflammatory biomarkers, even in the absence of a medical illness, and inflammatory illnesses are associated with greater rates of depression, suggesting inflammation is linked to depression [46].

#### Tryptophan metabolism can account for the etiology of these depressive states:

**Figure 1:**
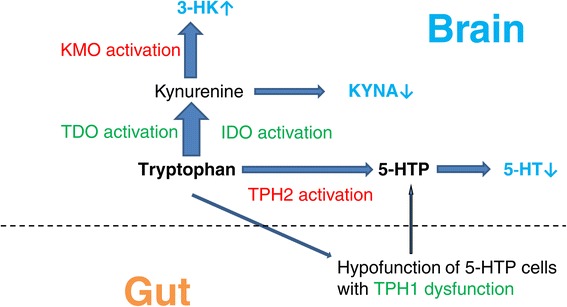
**Impaired glial-neuronal network.** Impaired glial-neuronal network expressed by peripheral and central key enzymes of tryptophan metabolism. Hypofunctional peripheral 5-HTP cells (reflecting TPH1 dysfunction) cause a reduction in 5-HTP levels that lead to a reduction in brain 5-HT levels. In major depression, neurotoxic kynurenine metabolites (e.g., 3-HK and QUIN derived from 3-HK) and/or TPH1 dysfunction combined with compensatory TPH2 activation, may induce astrocytic and neuronal apoptosis.

Reactive depression is related to dysregulation of the HTPA axis, a major component of the neuroendocrine system that controls stress reactions and regulates many body processes, mood and emotions, sexuality, and also energy storage. The HTPA axis is the node for interactions between the glands, hormones, and midbrain structures involved in the general adaptation syndrome. Cortisol produced by the adrenal glands activates tryptophan 2,3-dioxygenase (TDO) in the liver, thus increasing kynurenine synthesis [47-49] (Figure 1).

In secondary depression, inflammation, infection, or oxidative stress activate indoleamine 2,3-dioxygenase (IDO) in extrahepatic tissues, including the lungs, kidneys, spleen, blood, and brain [50,51], shifting tryptophan metabolism away from the liver [52]. Consequently, tryptophan breakdown through the kynurenine pathway occurs mainly in the blood and lymphoid tissues [53]. Because liver cell uptake of extrahepatic kynurenine is not efficient, metabolism mainly occurs extrahepatically. A key enzyme of the kynurenine pathway, IDO, and kynurenine-3-monooxygenase (KMO) that catalyzes 3-hydroxykynurenine (3-HK) production, are activated by proinflammatory cytokines including interleukin-1 and -6, tumor necrosis factor-α, and interferon-γ.

In both these depressive states, the brain kynurenine pathway is strongly activated from central and also peripheral kynurenine sources, as kynurenine can be transported across the BBB. In the brain, tryptophan catabolism occurs mainly in astrocytes and microglia [54-56]. Although some neurons also contain IDO and/or TDO [57], neurons are not the main sites of the kynurenine pathway within the brain. Neuroprotective KYNA and neurotoxic 3-HK are two intermediate metabolites of kynurenine. While astrocytes mainly produce KYNA because of a lack of the KMO enzyme, microglia and macrophages produce mainly the neurotoxin quinolinic acid (QUIN) from the 3-HK pathway [58-60], therefore the balance between 3-HK and KYNA may generally shift towards 3-HK and downstream neurotoxin production. It is possible that KMO activation is related to depression. Steiner *et al.* reported that region-specific increases in concentrations of QUIN, derived from 3-HK in the anterior midcingulate cortex and subgenual anterior cingulate cortex, may directly contribute to disturbed balance in glutamatergic throughput, potentially explaining the rapid response of severe depression to infusion of NMDA antagonists (e.g., ketamine) [61]. In major depression, there is evidence of neurodegenerative changes and loss of astrocytes. Neurotoxic kynurenine metabolites, such as 3-HK and QUIN, may induce astrocytic and neuronal apoptosis, weakening the glial-neuronal network responsible for induction of astrocytic neurotrophic factor synthesis, e.g., glial-derived neurotrophic factor (GDNF) and BDNF [62].

By shifting the equilibrium position of key enzymes in tryptophan metabolism (as described above), the classical depression classification can be reorganized as below:

**Peripheral classification of depression by key enzymes**

- TPH1 dysfunction
- TDO activation
- IDO activation

**Central classification of depression by key enzymes**

- TPH2 activation
- KMO activation

Among these key enzymes, only TPH2 activation (led by feedback from 5-HT depletion) provides alleviation from depression, partially compensated for by 5-HT synthesis. Enhanced tryptophan breakdown and further metabolism via the kynurenine pathway by both TDO and IDO, induces low tryptophan availability for 5-HT synthesis in the brain, further accelerating feedback of TPH2 activation. Conversely, TPH1 dysfunction, TDO activation, IDO activation, and KMO activation all exacerbate the depressive situation (Figure 1).

#### Metabolic depression and cybernetics

Metabolic depression, an adaptive biological process for energy preservation, is responsible for dormancy, torpor, hibernation, and estivation in animals [63]. From this point, depression in humans may be a purposive survival strategy, evident under threat of a metabolic energy crisis, and easily considered the default metabolic state until conditions improve following metabolic up-regulation as energy availability increases. A form of metabolic depression is assumed in the underlying observed hypometabolism, state-dependent neurobiological changes, and vegetative symptoms of major depression in humans. Metabolic depression is reactivated via differential gene expression in response to perceived adverse stimuli in predisposed individuals. The cellular (micro)environment in which the gene resides dominates the status quo. Consequential dormancy and major depression are characterized by withdrawal from the environment, lack of energy, loss of weight (from not eating and burning stored fat), and changes in sleep patterns. It is apparently not purposive for depressed patients to maintain or raise the metabolic rate to cope with decreased 5-HT precursor, the severity of life stresses, and a chronic inflammation state, hence they suppress metabolism and remain in torpor until conditions improve. There are considerations for the natural course of depression. Nowadays, it would not be permitted ethically to observe depressed patients without antidepressant treatment for an extended period of time. However, from a historical perspective, in 1921 before the discovery of antidepressants, Kraepelin speculated that in most cases, major depressive episodes tend to last about 6–8 months without treatment [64]. In short-term and mild depression, the depressive process is adaptive and rational, preventing other intestinal disturbances, life event stressors, and inflammations, by withdrawing from group activities due to depressed mood and/or loss of interest. However, in chronic and/or severe depression, the process may trigger a life-threatening pathological condition (e.g., impaired glial-neural network, thoughts of death or suicide, suicide plan, losing a job).

Norbert Wiener defined cybernetics from a Greek word meaning “the art of steering”, as “the scientific study of control and communication in the animal and the machine” [65]. It is applicable when a system is involved in a closed signaling loop, i.e., action by the system generates an environmental change that is reflected in the system in some manner (feedback), triggering a system change. Originally, this was referred to as a “circular causal” relationship. In applying the idea of cybernetics to depression, it is necessary to define and integrate the functions and processes of the depressive condition using key enzymes that can move from the environment to desired conditions or treatments.

The primary pivots steering the course of depression may be key enzymes involved in tryptophan metabolism. Potentially, all types of depression can be expressed through combinations of these key enzymes using a matrix with peripheral (TPH1, TDO, IDO) and central (TPH2, KMO) determinants. Moreover, this classification is not mutually exclusive, but adaptable and cumulative based on enzyme malfunction. For example, hypothetically, if a patient with endogenous depression was severely burned and his/her house burnt to ashes, losing all his/her fortune, this depressive condition could be expressed as a set of depressive states, incorporating peripheral (TPH1 dysfunction, TDO activation, IDO activation) and central (TPH2 activation, KMO activation) determinants.

From a therapeutic point of view, it is important to reset the shifting equilibrium position to a natural, balanced state. Treatment options from the etiological classification of depression may include gene therapy to rescue intestinal 5-HTP cells or enhance peripheral 5-HTP production and thereby compensate for TPH1 dysfunction, psychotherapy for TDO activation, and anti-inflammatory drugs for IDO activation. A goal of cybernetics for depression is steering key enzyme equilibrium positions to recover neutral metabolic conditions.

#### Comorbid medical/psychiatric conditions

A matrix with peripheral (TPH1, TDO, IDO) and central (TPH2, KMO) determinants may be considered the status quo for various comorbid medical/psychiatric conditions. For example, Cushing’s disease (in which the pituitary gland produces pathologically high ACTH levels, with ACTH signaling to the adrenal glands increasing cortisol production) and Cushing syndrome (caused by overuse of corticosteroid medications), increase vulnerability to depression because of peripheral TDO activation by cortisol. Conversely, drugs that suppress cortisol (e.g., cyproheptadine, sodium valproate, and bromocriptine) manage depressive states caused by peripheral TDO activation [66]. Patients with Crohn’s disease are often affected by depression*.* Crohn’s disease (also known as regional enteritis) is a type of inflammatory bowel disease that affects any part of the gastrointestinal tract from the mouth to the anus, and may be expressed as peripheral TPH1 dysfunction and IDO activation, induced by intestinal inflammation. Isoniazid is an organic compound that is the first-line medication for tuberculosis, and also a weak monoamine oxidase (MAO) inhibitor. The era of antidepressants started with this drug, which was accidentally found to have euphoric effects in patients with tuberculosis. Using this matrix, the effect of isoniazid may be expressed by IDO suppression (owing to reduced inflammation), central TPH2 suppression (via the MAO inhibitory effect, leading to negative feedback at the rate-limiting TPH2 step), and KMO suppression (owing to reduced inflammation, concomitant with IDO suppression). In this manner, the matrix of peripheral (TPH1, TDO, IDO) and central (TPH2, KMO) determinants can encompass comorbid medical/psychiatric conditions with a variety of symptoms and be a tool for thought experiments.

#### Testing the hypothesis

The technology to directly measure enzyme activity has not yet been developed, and the following experimental approaches are essential to empirically test TPH1 activities.

- Collecting (through biopsy or autopsy) and comparing intestinal 5-HTP cells from patients with major depression and a normal control group.
- Monitoring serum and urinary 5-HTP will be useful to match levels to different phases of major depression.

The second study will assume compensatory TPH2 activities for major depression through cerebrospinal fluid (CSF) levels of the 5-HT metabolite, 5-hydroxyindoleacetic acid.

The following experimental approaches are essential to empirically test TDO and IDO activities.

- Observing relative increases in plasma or salivary cortisol, and cytokine levels induced by inflammation, and determining the effect on subsequent depression development.

The second study will assume KMO activities for major depression through CSF QUIN levels.

To test this classification, it is necessary to further analyze genetic and enzymatic involvement, and examine consistency in diverse mental disorders. This classification can be regarded as provisional until it has been verified or disproved by satisfactory evidence of changes in metabolites, or ratios between metabolites, as biomarkers. If contradictory classifications are reported, this classification will be rejected.

## Summary

The linked tryptophan–5-HT and tryptophan–kynurenine pathways represent a major junction between gene-environment interactions. The described etiological classification expressed by peripheral (TPH1, TDO, IDO) and central (TPH2, KMO) enzymes allows depression to be viewed as a clear box with the inner components, or logic, available for appropriate individualized inspection and treatment.

## Abbreviations

TPH: Tryptophan hydroxylase
TDO: Tryptophan 2,3-dioxygenase
IDO: Indoleamine 2,3-dioxygenase
3-HK: 3-hydroxykynurenine
KYNA: Kynurenic acid
KMO: Kynurenine-3-monooxygenase
5-HT: Serotonin
BBB: Blood–brain barrier
BCAAs: Branched chain amino acids
EC: Enterochromaffin
5-HT cells: 5HT-producing cells
5-HTP cells: 5-hydroxytryptophan -producing cells
HTPA axis: Hypothalamic-pituitary-adrenal axis
QUIN: Quinolinic acid
GDNF: Glial-derived neurotrophic factor
BDNF: Brain derived neurotrophic factor
MAO: Monoamine oxidase
CSF: Cerebrospinal fluid
